# Deviations of rational choice: an integrative explanation of the endowment and several context effects

**DOI:** 10.1038/s41598-020-73181-2

**Published:** 2020-10-01

**Authors:** Joost Kruis, Gunter Maris, Maarten Marsman, Maria Bolsinova, Han L. J. van der Maas

**Affiliations:** 1 Psychological Methods Department, University of Amsterdam, Nieuwe Achtergracht 129B, Amsterdam, 1018WS The Netherlands; 2 ACT-Next by ACT, 500 ACT Drive, Iowa City, IA 52245 USA; 3 Department of Methodology and Statistics, Tilburg University, Warandelaan 2, Tilburg, 5037 AB The Netherlands

**Keywords:** Human behaviour, Statistics, Statistical physics

## Abstract

People’s choices are often found to be inconsistent with the assumptions of rational choice theory. Over time, several probabilistic models have been proposed that account for such deviations from rationality. However, these models have become increasingly complex and are often limited to particular choice phenomena. Here we introduce a network approach that explains a broad set of choice phenomena. We demonstrate that this approach can be used to compare different choice theories and integrates several choice mechanisms from established models. A basic setup implements bounded rationality, loss aversion, and inhibition in a natural fashion, which allows us to predict the occurrence of well-known choice phenomena, such as the endowment effect and the similarity, attraction, compromise, and phantom context effects. Our results show that this network approach provides a simple representation of complex choice behaviour, and can be used to gain a better understanding of how the many choice phenomena and key theoretical principles from different types of decision-making are connected.

## Introduction

The response behaviour of humans on (discrete) choice problems has been extensively studied in many fields of science, such as economics^1–4^, psychology^5–8^, psychometrics^9,10^, cognitive science^11–14^, neuroscience^15,16^, and engineering^17,18^. Traditional theories of choice assume the decision-maker as a *homo economicus*^19,20^, i.e., rational^1,5,21^. For choices to be rational all choice alternatives must be comparable and have transitive preference relations, so they can be ordered by the decision-maker. A second feature, and a central principle of rational choice theory, is that a rational decision-maker consistently chooses the outcome that maximises utility, or expected utility for risky or uncertain choices^5,22–24^. These assumptions clearly fail the scrutiny of everyday experience. To account for the observed inconsistencies, most models nowadays characterise choice as a probabilistic process^6,9,21,24–29^.

A prominent group of probabilistic choice models, such as Luce’s strict utility model^6,24^ and the multinomial logit model^21^ for preference, and Bock’s nominal categories model^30^ for aptitude, are characterised by the following distribution for the choices:

1 $$\begin{aligned} \begin{aligned} p_{\scriptscriptstyle {S}}(x)&= \frac{\exp {\left( \pi _x\right) }}{\sum \limits _{y \in \scriptscriptstyle {S}} \exp {\left( \pi _y\right) }} \, , \end{aligned} \end{aligned}$$

in which $p_S(x) \in [0,1]$ represents the probably of choosing alternative *x* from the set of possible alternatives *S* as a function of the utility of alternative *x*, $\exp (\pi _x)$, where $\pi _x \in \mathbb {R}$. This distribution is also known as the Boltzmann distribution^31,32^ from statistical mechanics. For binary choice problems $(S = \{x,y\})$ Eq. (1) takes a form known as the Bradley–Terry–Luce model in the decision-making literature^33,34^, or as the Rasch model^9^ in psychometrics:

2 $$\begin{aligned} \begin{aligned} p_{x,y}(x)&= \frac{\exp {\left( \pi _x\right) }}{\exp {\left( \pi _x\right) } + \exp {\left( \pi _y\right) }} = \frac{\exp {\left( \pi _x - \pi _y\right) }}{1 + \exp {\left( \pi _x - \pi _y\right) }} \, . \end{aligned} \end{aligned}$$

Models with this form have the property of simple scalability, which implies that the probability of choosing option *x* over option *y* is strictly increasing in the utility of *x* and strictly decreasing in the utility of *y*. Several properties with respect to independence from irrelevant alternatives (IIA) and transitivity follow from simple scalability. The degree to which choices are considered rational from a probabilistic perspective, is often described by the extent to which the choice probabilities for a set of choice alternatives possess these properties^6,24,35–37^.

For example, the weakest form of IIA is regularity, which implies that the probability of choosing an alternative can never increase by adding more alternatives. A set of preference probabilities is regular if $x \in A \subseteq S$ and $p_{\scriptscriptstyle {A}}(x) \ge p_{\scriptscriptstyle {S}}(x)$. The strongest form of IIA is the choice axiom that is satisfied when $x \in A \subseteq S$ and $p_{\scriptscriptstyle {S}}(x) = p_{\scriptscriptstyle {A}}(x) \sum _{y \in {\scriptscriptstyle {A}}}p_{\scriptscriptstyle {S}}(y)$. Meaning that the probability of choosing an alternative from a particular set is equal to the probability of choosing the alternative from a subset times the probability of selecting any alternative in this subset from the original set. Models are characterised as either strict, strong, or weak binary utility models depending on the expression that can be used to obtain the binary choice probabilities. If the positive real number $v_i$ denotes the utility of alternative *i*, then a model is strict if $p_{x,y}(x) = v_x/(v_x + v_y)$, strong if $\phi$ is a cumulative distribution function with $p_{x,y}(x) = \phi [v_x - v_y]$ and $\phi [v_x - v_x] = {}^{1}\!/_{2}$, and weak if $p_{x,y}(x) \ge {}^{1}\!/_{2}$ when $v_x \ge v_y$. Rationality is also assessed by considering different observable properties of pairwise probabilities, such as the product rule, quadruple condition, strong, moderate, and weak stochastic transitivity, and the multiplicative and triangle conditions, each describing a different degree of strictness in the ordering of the choice probabilities. We refer the reader to Luce and Suppes^24^ for a comprehensive treatment of these properties.

Although models with simple scalability have statistically desirable properties, their assumptions are often violated in reality. In the preferential choice literature for example, violations of IIA known as the similarity, attraction, compromise, and phantom context effects describe different situations in which the preference relation between two choice alternatives changes when a third alternative is introduced^7, 24,38–52^. Another example is the endowment effect that describes the tendency of people to perceive an alternative as having increased in value after they have chosen it^3^. We will discuss these violations and phenomena in more detail later.

Over time, theories and models have been adapted or extended to account for these deviations. Bounded rationality^53^, for example, is the theory that instead of searching for the alternative with maximum utility, we search until we find the first alternative with satisfactory utility. Loss aversion, which postulates that the perceived utility of not losing something is greater than the perceived utility of gaining that exact same thing, was offered as an explanation for the endowment effect^3,54,55^. Whereas the elimination by aspects model^42^ provided a first account for the similarity effect only, multi-attribute multi-alternative sequential sampling models, such as multi-alternative decision field theory^14,56–60^, the leaky competing accumulator model^61–64^, the multi-attribute linear ballistic accumulator model^65–67^, the $2N-$ary choice tree model^68,69^, and the associations and accumulation model^70^, also account for the attraction and compromise context effect using a range of different mechanisms. The interested reader is referred to three recent papers that offer a comprehensive comparison between the different models^37,71,72^.

Although these approaches are capable of modelling context effects, one drawback is that they are fairly complex. With increasing complexity generalisability often takes a hit, and models that are tuned to account for one type of choice effect fail to account for other choice effects, hence drawing inferences beyond the task-setting becomes challenging. Developing a simple choice model that is capable of connecting a broader spectrum of choice phenomena would thus be a worthwhile effort. For one, unifying distinct phenomena in a collective framework puts them on equal footing and hence can stimulate the development of formal theories that can account for all of them. Also, formalising our theories requires us to be precise and concrete, this in contrast to verbally formulated theories which are easily misinterpreted, often hold hidden assumptions, claim predictions that are not clearly derived from the theory, as well as hide consequences from the model that are not desired. Moreover, formal theories may lead to interesting predictions and new insights, and hence new possibilities to falsify the theory, that might not have been discovered if the phenomena are investigated independently. In this paper we propose such a probabilistic model for choices that conceptualises choice problems as a combination of a choice structure, an alternative evaluation process, and a choice trigger condition.

In the remainder of this paper, we start by introducing the choice structure, represented by a network in which the nodes are the cues and alternatives, and the edges describe the relationships between them. We demonstrate a basic setup of our choice model, in which the binary node states follow a distribution known as the quadratic exponential binary distribution, or Ising model, and alternatives are evaluated with single spin-flip dynamics. Sampling choices from the invariant distribution of the configurations in which only one alternative is active, the choice conditions, gives us choice probabilities with the same form as the Boltzmann distribution from Eq. (1). Triggering a choice as soon as the condition holds for the first time, implements bounded rationality and predicts the occurrence of context effects. We then discuss how our approach compares to multi-attribute multi-alternative models and implements, or might be extended with, the mechanics used by those models. Finally, we discuss some challenges and future directions for the model.

## Results

Here we introduce the different components of the choice model and derive predictions for choice probabilities and response times.

### Choice model

The choice model consists of a structure, a process, and a trigger. The choice structure describes the alternatives available for a choice and the origin of their utilities. The choice process describes how the alternatives are evaluated. The choice trigger describes the condition that stops the evaluation process and prompts a decision.

The specific form of these three components allows for some variation depending on the specific setting. For example, in this paper we let the state of cues and alternatives in the choice structure be either active or inactive. While this is reasonable in the case of preferential choice, in the case of modelling an opinion we might want to use three possible states, namely pro, neutral, or against. These types of variations are also possible in the case of the process and trigger elements of the choice model, and we discuss several of them throughout the paper.

#### Structure

**Figure 1:**
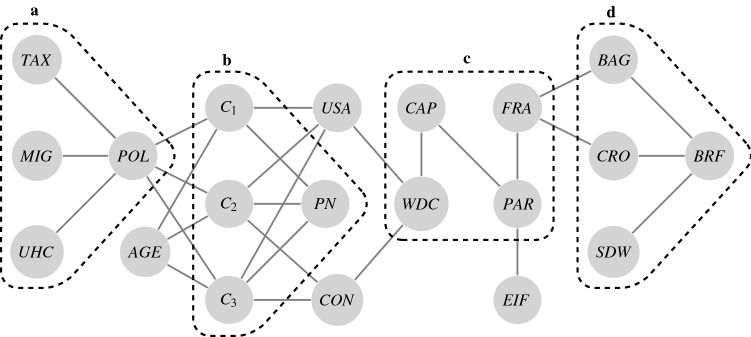
Graph of (related) concepts and subsets of concepts as possible choice problems. Nodes represent concepts and edges represent a relationship between concepts. Nodes surrounded by a dashed box, represent concepts that can form a potential choice problem. For example, (**a**) If you have to choose between taxes (*TAX*), migration (*MIG*), or universal health care (*UHC*), which policy (*POL*) is most important for you? (**b**) Do you prefer Candidate 1 $(C_1)$, Candidate 2 $(C_2)$ or Candidate 3 $(C_3)$, as the Presidential Nominee (*PN*)? (**c**) Is Washington DC (*WDC*) or Paris (*PAR*) the capital (*CAP*) of France (*FRA*)? (**d**) Do you want a sandwich (*SDW*), a baguette (*BAG*), or a croissant (*CRO*) for breakfast (*BRF*)? Concepts present in the graph but not part of a subset are respectively, age (*AGE*), United States of America (*USA*), Congress (*CON*), and Eiffel Tower (*EIF*).

In their simplest form choices can be structured as a combination of cues and alternatives and the relationships between them. Cues represent the conditions of the choice, e.g., ‘buy a book’, ‘select a present’, or ‘solve for *x*’, and alternatives describe the possible choices. An appropriate representation of such a structure is a network in which the nodes correspond to the alternatives and the cues, and the edge between two nodes describes their relation. Figure 1 shows how the structure of a particular choice problem can be seen as a subset from a larger collection of related concepts.

**Figure 2:**
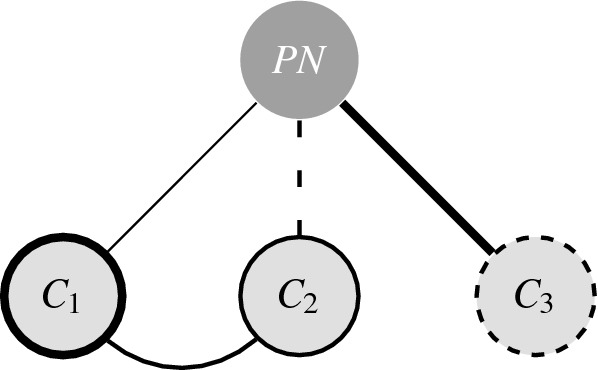
Choice structure with a single cue (*PN*) and three alternatives $(C_1, C_2, C_3)$. Cues are represented as dark grey nodes with white text and alternatives are represented as light grey nodes with black text. Edges represent a positive (solid) or negative (dashed) relationship between nodes, and a ring around a node represents whether the nodes is generally appealing (solid) or unappealing (dashed). The thickness of both the edges and rings around the nodes corresponds to intensity of the relationship/appeal.

To arrive at predictions about choice behaviour we assume that both the type and strength of a relationship between two nodes can vary, and that nodes outside of the choice subset can also influence a decision through their relationship with nodes that are in the choice subset. In Fig. 2 possible relationships between a cue and the alternatives are illustrated for the choice structure from Fig. 1b.

We refer to the experienced magnitude and direction of an alternative’s utility in terms of an alternative’s appeal. Figure 2 shows that an alternative’s appeal is a function of its general appeal and relationship with the cue and the other alternatives. The general appeal of an alternative captures the relation between the alternative and nodes that are not in the choice structure. For example, in Fig. 1 we see that the general appeal of a candidate is a function of policy and age. The relation with a cue can positively or negatively affect the appeal of an alternative. For example, asking *Do you want a nice and fresh croissant, yesterdays leftover sandwich, or a somewhat dry baguette, for breakfast?* enhances the appeal of the croissant through the suggestive phrasing of the cue. A relation between two alternatives signals that the appeal of one is related to that of the other alternative. The next step is formalising the choice structure as a probability distribution.

For a choice structure with *n* nodes, let $\mathbf {x} = [x_1, x_2, \dots , x_n]$ be a vector representing the configuration of the node states in which $x_i \in \{0,1\}$ denotes whether node *i* is active $(x_i = 1)$ or inactive $(x_i = 0)$. Let $\mathbf {A}$ be a symmetric $n \times n$ matrix in which $a_{ij} \in \mathbb {R}$ describes the relation between the node *i* and node *j* in the choice structure. Let $\mathbf {b} = [b_1, b_2, \dots , b_n]$ be a vector of length *n* in which $b_i \in \mathbb {R}$ describes the general appeal of node *i*. A valid probability distribution over the states is obtained by endowing them with the following distribution:

3 $$\begin{aligned} \begin{aligned} \mathbf {x} \sim p(\mathbf {x})&= \frac{1}{Z} \exp {\left( {\textstyle {\beta }} \left[ \sum \limits _{\langle i,j \rangle }a_{ij} \, x_i x_j + \mu \sum \limits _i b_i \, x_i \right] \right) } \, , \end{aligned} \end{aligned}$$

in which $\beta$, a non-negative real number, and $\mu \in \mathbb {R}$ are scaling constants, $\sum _{\langle i,j \rangle }$ denotes the summation over all distinct pairs of *i* and *j*, and *Z* is the normalising constant that sums over all the $2^n$ possible configurations of $\mathbf {x}$ such that the probabilities of the possible states sum to one. We can multiply $\beta$ by some constant and divide $\mathbf {A}$ and $\mathbf {b}$ by the same constant without affecting the probabilities of the states, the same holds for $\mu$ and $\mathbf {b}$. As such, we set both $\beta$ and $\mu$ to one for now, making them drop out of the equation, and discuss later how they might be used to model choice setting variations, such as time-pressure and/or individual differences.

The distribution in Eq. (3) can be recognised as the Ising model^73,74^, a highly popular and one of the most studied models in modern statistical physics^75^, or as the *quadratic exponential binary distribution* as it is known in the statistics literature^76,77^. Capable of capturing complex phenomena by modelling the joint distribution of binary variables as a function of main effects and pairwise interactions^78^, it has been used in fields such as genetics^79^, educational measurement^80^, and psychology^78,81–83^. In the context of choice it has been applied in sociology in Galam’s work on group decisions in binary choice problems^84,85^. In this application each node represents the choice of one person on a specific problem, and the pairwise interactions describe the influence of all people in the group on the individuals choice. Another application is the Ising Decision Maker from Verdonck and Tuerlinckx^86^, a sequential sampling model for speeded two-choice decision-making. In this model each of the two alternatives is represented by a pool of nodes, inside a pool nodes excite each other, between pools nodes inhibit each other. A stimulus is represented by a change in the external field, after which the node states are sequentially updated. The response process monitors the mean activity per pool, and chooses the first alternative for which this activity crosses a threshold. Both these models use this distribution in a substantially different way compared to the current application, and have not been applied to explain deviations from rationality. As such we will not discuss them in more detail for this paper.

A connection between Eq. (3) and probabilistic choice models is found by realising that the distribution of $\mathbf {x}$ is a function of the Hamiltonian:

4 $$\begin{aligned} \begin{aligned} H_{\mathbf {x}}&= - \sum \limits _{\langle i,j \rangle }a_{ij} \, x_i x_j - \sum \limits _i b_i \, x_i \, , \end{aligned} \end{aligned}$$

and that the probability of each configuration is given by plugging $H_{\mathbf {x}}$ in the Boltzmann distribution from Eq. (1). That is, if *S* is the set of all configurations that a particular system can take and $\mathbf {x}$ is one possible configuration of this system, then the probability of the system being in this state is given by:

5 $$\begin{aligned} \begin{aligned} p_{\scriptscriptstyle {S}}(\mathbf {x})&= \frac{\exp \left[ - H_{\mathbf {x}}\right] }{\sum \limits _{\mathbf {y} \in {\scriptscriptstyle {S}}} \exp \left[ - H_{\mathbf {y}}\right] } \, . \end{aligned} \end{aligned}$$

We assume that until a person is faced with a choice, the internal state of the decision-maker (the resting configuration) is distributed according to Eq. (3). An advantage of this assumption is that well defined stochastic processes for these systems exist and can be used in the next component of the choice model that describes how alternatives are evaluated until a choice is triggered. When a person is confronted with a choice all cue nodes are activated and remain so during the choice process. The alternatives will, in most cases, be distributed according to the resting state distribution. Exceptions to this are discussed later on.

#### Process

Although many configurations for the choice process are possible, to illustrate our approach we use a simple stochastic process for interacting particle systems to model the process of alternative evaluation. Specifically, a Metropolis algorithm with single spin-flip dynamics^87^ in which a proposal configuration is generated at each iteration by sampling one alternative and flipping its state:

1. Let $\mathbf {x}$ denote the current configuration of the system with $H_{\mathbf {x}}$
2. Select one node *i* at random and flip its value $x^*_i = 1 - x_i$
3. Calculate $H_{\mathbf {x}^*}$ for the configuration with the flipped node.
4. If $H_{\mathbf {x}^*} < H_{\mathbf {x}}$, keep the configuration with the flipped node.
5. If $H_{\mathbf {x}^*} \ge H_{\mathbf {x}}$, keep the configuration with flipped node with probability $\exp {\left( H_{\mathbf {x}} - H_{\mathbf {x}^*}\right) }$.

For a choice with *m* alternatives the evaluation process will thus transition between $2^m$ possible configurations of the alternative states.

#### Decision

From Eq. (4) it can be derived that in a choice structure in which both the general appeal and the relationships are positive, the most likely configuration is the one with all alternatives active. This is reasonable as it implies that the most preferred state for a decision-maker is to posses all alternatives. In most applications a person is forced to choose only one of the alternatives, however. We impose this by defining potential choice conditions as configurations in which only a single alternative is active and discuss two possibilities for making decisions.

The first is that the alternative evaluation process is terminated when the single-spin flip algorithm has converged and a choice is sampled from the invariant distribution of the potential choice configurations:

6 $$\begin{aligned} \begin{aligned} p\left( x_i \, {\textstyle {\in \, M}} = 1 \mid \sum \limits _{j = 1}^{m + k} x_j = k + 1\right)&= \frac{\exp {\left( \sum \limits _{k \in {\scriptscriptstyle {K}}} a_{ik} + b_i \right) }}{\sum \limits _{m \in {\scriptscriptstyle {M}}} \exp {\left( \sum \limits _{k \in {\scriptscriptstyle {K}}} a_{mk} + b_m \right) }} \, , \end{aligned} \end{aligned}$$

in which $M = [x_1, x_2, \dots , x_m]$ denotes the subset of *m* alternative nodes and $K = [x_{m + 1}, x_{m + 2}, \dots , x_{m+k}]$ denotes the subset of *k* cue nodes. If we let the Markov chain run until convergence, the effect of any interactions between choice alternatives will have worn out and the property of simple scalability will hold for the choice probabilities, guaranteeing that choices are in accordance with the choice axiom. The choice axiom is known to be violated in particular choice problems, however, which leads us to the second choice trigger possibility.

At some moment during the process a potential choice condition is met for the first time. One could say that a choice has effectively been made and there is no need for a decision-maker to continue. This choice trigger implements the idea of bounded rationality and explains various types of irrational choices as we explain after we discuss the consequences of our model setup for rational choices.

### Rational choice

Although our setup implements bounded rationality, it does not preclude rational choices. However, while choice structures can be made for which even the strongest gradation of rationality holds, finding clear cut rules for when a structure adheres to which gradations of rationality is a different kettle of fish. In the methods section we show that a very simple expression exists for the expected choice probabilities in the single spin-flip algorithm as a function of the transition matrix for the possible configurations of the alternatives. Deriving general rules for the adherence to different types of rationality requires one to express these probabilities as a function of the parameters $\mathbf {A}$ and $\mathbf {b}$. As this expression is already of a gargantuan size for $n=3$, and there is no reasonable way to derive general algebraic properties from it, we only work out the binary case in the methods section and show that even then determining when choices are guaranteed to be at least weakly rational is not necessarily straightforward.

For $n>2$ the expectation of rational behaviour for a particular choice structure has to be derived on a case by case basis. As for *n* alternatives there are $2^n - n - 1$ possible subsets of at least two variables, investigating the assumption of independence of irrelevant alternatives will be more time consuming compared to determining properties of the pairwise probabilities of a choice set. A statistical program such as R^88^ can calculate these expected pairwise choice probabilities in reasonable time for choice situations with up to 15 alternatives using the expression from the methods section. For larger numbers of alternatives numerical solutions can be obtained with a simulation approach. Additionally, assumptions that simplify the analytical expression for the expected choice probabilities can also be used to derive rational choice properties.

### Irrational choice

We define irrational decision-making as those choice situations in which the odds of choosing one alternative over the other, as established by their pairwise choice probabilities, changes as a function of adding other alternatives to the set. We realise that for readers well versed within the choice literature this definition may seem both rather vague, because our definition creates a dividing line somewhere between the choice axiom and regularity, as well as strict, as violating the choice axiom means that the strictest rules and conditions for rationality can still hold for the binary choice probabilities. However, although we touched upon the different gradations of rationality in the previous paragraphs, we think that a more conceptual approach is more appropriate here. We will discuss examples in which it is immediately clear that the choice probabilities as predicted by rational choice theory are conceptually counter intuitive.

Context effects are perhaps the most well known and studied violations of IIA and are often described by a situation in which a preference relation between two alternatives, a target and a rival, is established. Then a third alternative is introduced, the decoy, and it is demonstrated that adding the decoy changes the choice probabilities in favour of the target. These effects can range from only increasing the probability for the target while keeping the original order of the preference relations between the alternatives intact, to a full reversal of the preference relation. In our model these effects can be explained by the presence of a relationship between two choice alternatives and its influence on the resting state distribution and the alternative evaluation process.

For several types of context effects we provide an example and show how it can be explained in our model. As our explanation of the context effect does not require bias in the presentation of the choice, we assume the relationship between all pairs of a cue and an alternative to be the same across the board $(a_{mk} = 1)$. In the Supplementary Materials we work out the specific steps to calculate the choice probabilities for our example of the attraction effect, as well as provide the parameter values for the other examples.

#### Similarity

The similarity effect^38,39^ describes the situation in which adding a decoy that is highly similar to the rival results in an increased preference for a dissimilar target alternative. The classic example for this effect was given as a thought experiment that provides the choice probabilities, expected under rational choice theory for a choice between three recordings:

“Let the set *U* have the following three elements:

- $D_C$, a recording of the Debussy quartet by the *C* quartet.
- $B_F$, a recording of the eighth symphony of Beethoven by the *B* orchestra conducted by *F*.
- $B_K$, a recording of the eighth symphony of Beethoven by the *B* orchestra conducted by *K*.

The subject will be presented with a subset of *U*, will be asked to choose an element in that subset, and will listen to the recording he has chosen. When presented with $\{D_C, B_F\}$ he chooses $D_C$ with probability ${}^{3}\!/_{5}$. When presented with $\{B_F, B_K\}$ he chooses $B_F$ with probability ${}^{1}\!/_{2}$. When presented with $\{D_C, B_K\}$ he chooses $D_C$ with probability ${}^{3}\!/_{5}$. What happens if he is presented with $\{D_C, B_F, B_K\}$? ...He must choose $D_C$ with probability ${}^{3}\!/_{7}$. Thus if he can choose between $D_C$ and $B_F$, he would rather have Debussy. However, if he can choose between $D_C$, $B_F$, and $B_K$, while being indifferent between $B_F$ and $B_K$, he would rather have Beethoven.”    Debreu, 1960^38^.

It is clear that these expected choice probabilities are highly implausible. Specifically, in this case one would expect that when presented with $\{D_C, B_F, B_K\}$, $D_C$ would be chosen with probability ${}^{3}\!/_{5}$ and the remaining ${}^{2}\!/_{5}$ would be split evenly among $B_F$ and $B_K$. Such intuition has been proven correct in studies with a similar format as the thought experiment^7,41,42^.

**Figure 3:**
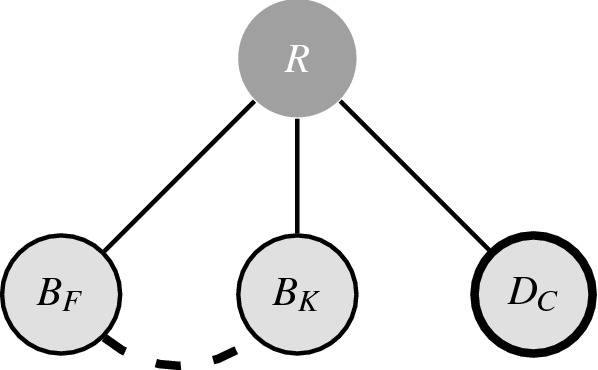
Choice structure for Debreu’s example of the similarity effect. With cue ‘choose a recording’ (*R*), and alternatives, ‘Beethoven conducted by *F*’ $(B_F)$, ‘Beethoven conducted by *K*’ $(B_K)$, and ‘Debussy by the *C* quartet’ $(D_C)$.

One choice structure that explains the similarity effect does this by introducing a negative association between the two Beethoven recordings, as in shown in Fig. 3. The negative relation between $B_F$ and $B_K$ has no influence on choice probabilities for any of the possible two-element subsets, as such the slightly larger base appeal of $D_C$ will result in choosing $D_C$ with probability ${}^{3}\!/_{5}$ when presented with $\{D_C, B_F\}$ or $\{D_C, B_K\}$. When presented with $\{B_F, B_K\}$, the negative relation works in both ways and $B_F$ and $B_K$ are chosen with equal probability. While the conditional distribution of the model from Eq. (6) predicts that $D_C$ will be chosen with probability ${}^{3}\!/_{7}$ when a choice has to be made from all three alternatives together, the rule that one stops as soon as the choice conditions hold for the first time will actually predict that when presented with $\{D_C, B_F, B_K\}$, $D_C$ is chosen with probability ${}^{3}\!/_{5}$ and $B_F$ and $B_K$ are both chosen with probability ${}^{1}\!/_{5}$. Our explanation of the ‘irrational’ (yet intuitive) choice behaviour in this example of the similarity effect rests on the presence of a negative relation between the Beethoven recordings.

One could argue that the ability to reverse engineer a network structure until the desired choice probabilities are obtained is a weakness of our approach. We believe that this is actually an advantage as, for one, it is possible to check if adaptations of the choice structure will still result in plausible choice behaviour. For example, imagine that you chose $$B_K$$ from the set $$\{D_C, B_F, B_K\}$$ and are asked to choose once more from the remaining recordings $$\{D_C, B_F\}$$. Taking into account that you already have $$B_K$$$$(x_{B_K} = 1)$$, the negative relation between $$B_K$$ and $$B_F$$ in our choice structure results in a prediction that you will choose $$D_C$$ with near certainty. This demonstrates that the choice structure does not only explain observed behaviour, but also predicts new, and in this case plausible, behaviour for adaptations of the choice problem. Furthermore, as we will discuss in the next example, it also allows one to come up with theoretically distinct choice structures for a single choice phenomenon and compare them. While the initially expected choice probabilities might be the same, manipulations that result in distinct predictions for each choice structure can be tested.

#### Attraction

The attraction, or asymmetric dominance, effect^44,45^ describes the situation in which the addition of a decoy alternative that is a substandard version of the target increases the preference for the target. Simonson and Tversky^46^ investigated this effect by offering two groups a choice between (a subset of) 6 dollar $({\$})$, a nice pen $(P_+)$, and a (less attractive) plain pen $(P_-)$. In the first group, choosing from the subset $\{{\$}, P_+\}$, more people chose the money (64%) compared to the nice pen (36%). In the second group, choosing from the set $\{{\$}, P_+, P_-\}$, as expected, almost no one chose the plain pen (2%), however, the money was now only chosen 52% of the time, while the proportion of people choosing the nice pen rose to 46%.

**Figure 4:**
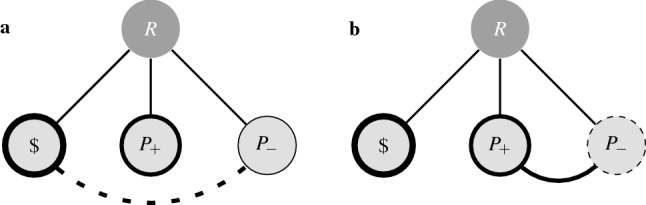
Choice structure for Simonson & Tversky’s example of the attraction effect. With cue ‘choose an Reward’ (*R*), and alternatives, ‘money’ $({\$})$, ‘nice pen’ $(P_+)$, and ‘plain pen’ $(P_-)$. The attraction effect can be explained with a negative relationship between the money and the plain pen (**a**), or a positive relationship between the two pens (**b**).

Figure 4 shows two possible choice structures that predict expected choice frequencies similar to those found in the experiment, however, each of these explain the attraction effect in a different way. In Fig. 4a the explanation of the attraction effect rests on the presence of a negative association between the money and the plain pen, while in Fig. 4b the effect is explained by a positive association between both of the pens. Our model thus provides two theoretically distinct choice structures that both explain how the mere addition of a less appealing decoy can boost the choice probabilities for the otherwise less frequently chosen target alternative.

Obtaining the same results from different structures allows us to compare the different theories and predictions that characterise each structure. For example, someone who chose $$P_+$$ as reward from the set $$\{{\$}, P_+, P_-\}$$ is asked to choose once more from the remaining rewards $$\{{\$}, P_-\}$$. Taking into account that this person already possesses $$P_+$$$$(x_{P_+} = 1)$$, the negative relation between $${\$}$$ and $$P_-$$, in choice structure **a**, results in a prediction of choosing $${\$}$$ in more than 90% of the cases, whereas the positive relation between $$P_+$$ and $$P_-$$, in choice structure **b**, results in a prediction of still choosing $$P_-$$ in 33% of the cases. While the initially expected probability distribution for choice structures a and b are thus the same, from the different prediction they make about a new situation, we can clearly distinguish which structure seems more plausible. The fact that our approach allows for these kinds of comparisons, and provides testable predictions, makes the theories captured in the model falsifiable.

#### Repulsion

In some cases the addition of a substandard version of the target alternative actually decreases the probability of selecting the target^89–92^. This reversed attraction effect, called the negative attraction or repulsion effect, although not consistently demonstrated, is mostly observed when choices are framed such that the decoy highlights the shortcomings of the more similar target alternative. For example, adding a smaller clementine to the choice between a fruit flavoured candy bar and an orange, might boost the probability of choosing the orange, as the clementine highlights the freshness and health aspects of citrus fruits. However, if the clementine shows some signs of a reduced freshness, e.g. crumpled skin or beginning to mould, it highlights the fleeting freshness of citrus fruit, and might instead boost the probability for the sugar filled candy bars and their long shelf life.

Just as the repulsion effect is the opposite of the attraction effect, so is its explanation, i.e., a positive relation between the rival and decoy alternatives. In the pen example from Fig. 4, switching the sign of the relation between the money $({\$})$ and the plain pen $(P_-)$ so it becomes positive, while keeping all other parameters the same, predicts a boost in the probability of choosing of the money $({\$})$ with respect to the nice pen $(P_+)$. Interestingly, whereas the negative relation in the attraction effect can result in a relatively large gain in choice probability for the target $(+ 10\%)$, the same structure but with a positive relation results in only a modest gain in the predicted choice probability for the rival $(+ 2\%)$. To increase the magnitude of the repulsion effect one has to decrease the general appeal of the added decoy. Finally, adding both an attracting and a repulsing decoy results in the context effects cancelling each other out when choosing between all four options.

#### Compromise

The compromise effect^45^ describes the situation in which a decoy is added for which the distance to the target mirrors that of the distance between the rival and the target, but in the opposite direction. This boosts the preference for the target alternative by making it seem like the compromise. Distance should in this context be interpreted as the relative position of the alternatives on particular attributes, such as prize and quality in the next example.

Tversky and Simonson^47^ investigated the compromise by offering two groups a choice between (a subset of) cameras of either low (*L*), medium (*M*), or high (*H*), prize and quality. While in the first group, choosing from the subset $\{L, M\}$, people chose both cameras with approximately equal probability, in the second group, choosing from the set $\{L, M, H\}$, people now chose both cameras *L* and *H* each with a probability of approximately ${}^{1}\!/_{4}$, while camera *M* was still chosen with a probability of approximately ${}^{1}\!/_{2}$. Based on the seemingly equal appeal of the *L* and *M* camera in group one, one would expect that both would be also be chosen with an approximately equal probability in group two. Or conversely based on the lower appeal of camera *L* in group 2, one would expect that in group one camera *L* would be chosen with a probability of approximately ${}^{1}\!/_{3}$ and hence camera *M* with a probability of approximately ${}^{2}\!/_{3}$.

**Figure 5:**
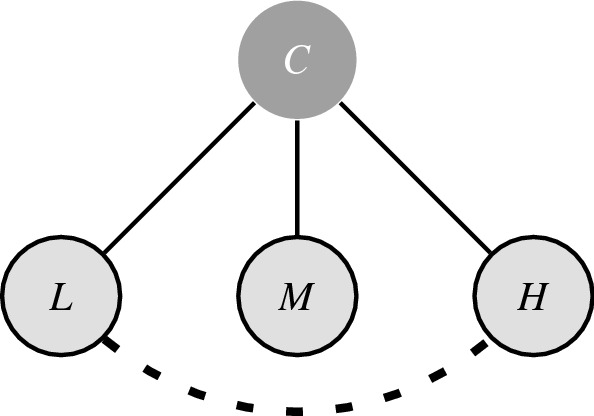
Choice structure for Tversky & Simonson’s example of the compromise effect. With cue ‘buy a Camera’ (*C*), and alternatives with respective quality and prize levels, ‘Low’ (*L*), ‘Medium’ (*M*), and ‘High’ (*H*).

A possible explanation for why this is not the case might be that the (dis)advantages between the cameras *H* and *L* camera are much more evident than those between the cameras *M* and *L* or *M* and *H*. Therefore, the weakness of camera *L* gets highlighted when camera *H* is part of the choice set, this in turn frames the camera *M* as the compromise that is of higher quality compared to camera *L*, but not as expensive as camera *H*. Once again, as is shown in Fig. 5, our explanation of the compromise effect can be captured by introducing a negative relation between the rival camera *L* and the decoy camera *H*.

So far the similarity, attraction, and compromise effect are each explained in our model by a negative interaction between the decoy and the rival. Whereas in the similarity effect, this relation is assumed to exist because of the large similarities between the rival and decoy alternatives, in the attraction and compromise effects, however, this relation is a function of the large dissimilarities between the two.

One explanation for this could be that only when (dis)similarities go into the extreme they are highlighted and start influencing the choice process. Another explanation comes from observed correlations between context effects, i.e., one study found that people who show the attraction effect also show the compromise effect, but not the similarity effect^60^. This could suggest that people either focus on similarities or dissimilarities, and hence the choice structure of a person only contains negative relations for one of those types. Whereas the attraction and compromise effect occur when a choice structure contains only negative relations as a function of dissimilarity, a choice structure in which negative relations are the result of similarity will only elicit the similarity effect. Not all context effects can be explained by a (negative) relation between the rival and decoy alternatives alone. In some cases it also manifests itself through the influence of the choice structure on the initial alternative configuration.

#### Phantom

The phantom decoy effect^52^ describes the situation in which the added decoy alternative is superior to both the target and rival alternatives, yet more similar to the target compared to the rival, but most importantly, unavailable. When it is communicated that the decoy cannot be chosen it subsequently boosts the preference for the target alternative.

Pratkanis and Farquhar^52^ studied the phantom decoy effect by offering two groups a choice between (a subset of) paperclips each with varying degrees of friction and flexibility. The target paperclip (*T*) and the rival paperclip (*R*), although different in these properties, were of comparable quality. The decoy paperclip (*D*) had a quality superior to both *T* and *R* but was in terms of friction and flexibility more alike to paperclip *T*. In the first group, choosing from the subset $\{T, R\}$, people chose each paperclip with approximately equal probability. People in the second group, however, who thought they where choosing from the set $\{T, R, D\}$, chose the paperclip of type *T* with a probability of approximately ${}^{4}\!/_{5}$, after the decoy *D* was revealed to be unavailable and hence the choice had to made again from the subset $\{T, R\}$.

**Figure 6:**
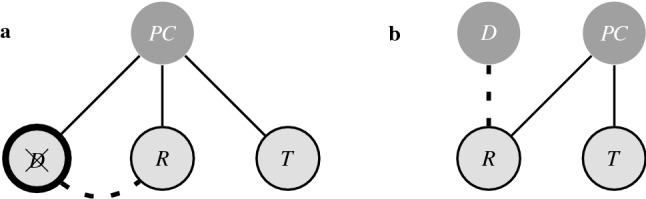
Choice structure for Pratkanis & Farquhar’s example of the phantom decoy effect. With cue ‘choose a Paper Clip’ (*PC*), and the decoy (*D*), rival (*R*), and target (*T*) paperclip alternatives. Depending on when the unavailability of the decoy is communicated, the phantom decoy effect is explained by a constrained version of the regular choice process (**a**), or an additional choice process in which the decoy is an extra cue (**b**).

As is shown in Fig. 6, our explanation of the phantom decoy effect, at this point perhaps unsurprisingly, partially rests on the presence of a negative relation between the rival and the decoy. It depends however, on when the unavailability of the decoy is communicated how the phantom effect is elicited. If this is communicated before the choice is offered the first time, the choice process is still updated to still sample and flip, but not terminate at, paperclip *D*. As shown in Fig. 6a, the combination of a negative relation between the *D* and *R* paperclips, together with the larger general appeal of paperclip *D*, reduces the probability for choosing paperclip *R*. If the unavailability of paperclip *D* is not communicated before the first choice and all three paperclips appear to be available, the choice structure from Fig. 6a without the previously introduced constrained will be evaluated and paperclip *D* is most likely to be chosen. At this point, the configuration of the choice structure is known, as only the cue and the node for paperclip *D* will be active. If at this point one is informed that paperclip *D* is unavailable, the choice process starts again from the known configuration. Given that node *D* is active, we can from this moment regard it as an additional cue, as is shown in Fig. 6b. Consequently, due to the negative interaction between paperclip *D* and paperclip *R*, flipping the *R* node and hence choosing it become less likely compared to paperclip *T*.

As shown in the Supplementary Materials, eliciting the phantom effect requires a much stronger negative relation between the decoy and rival when the unavailability of the decoy is known upfront, compared to when the unavailability is communicated after a choice is made for the first time. While it is easily argued that this is a rather intuitive hypothesis, it once again shows that our approach allows for making diverging predictions based on variations in the model setup.

#### Endowment

The endowment effect^3^ describes the situation in which people value an object higher if they possess it compared to when they do not. To illustrate this effect we consider a variation on the Debreu example in which you are given a Beethoven recording (*B*) and are immediately asked if you want to exchange it for an equally appealing Debussy recording (*D*). While the choice axiom predicts that you would exchange Beethoven for Debussy about half the time, the endowment effect says that people are unlikely to switch, a prediction that has been experimentally verified^93^. The endowment effect has been explained with choice-supportive bias^94^ and loss aversion^54^.

In our model both explanations would translate to an increase in base appeal of an alternative as soon as it has been chosen. With our setup we obtain a new explanation that does not depend on changes in the values of the choice problem but ties into the choice process itself. Having been given the Beethoven makes the choice conditions satisfied, and hence the initial configuration of the alternatives is known when offered to exchange it for the Debussy. Exchanging them requires a sequence of events in the choice process that, due to the equal appeal of both alternatives, has a lower probability compared to keeping the Beethoven. Specifically, the only way that switching becomes an option is when the initial state, the choice condition for the Beethoven, is left in the first iteration by sampling and accepting the flip of either node *B* or node *D*. From the resulting configurations both choices are then equally likely. Let $u_R = a_{RB} + b_B = a_{RD} + b_D$ denote the appeal for both the Beethoven and Debussy recordings. The probability of exchanging *B* for *D* is then given by:

7 $$\begin{aligned} \begin{aligned} p(D) = \frac{1}{2} \left[ p\left( \underset{\scriptscriptstyle {0 \rightarrow 1}}{D}\right) + p\left( \underset{\scriptscriptstyle {1 \rightarrow 0}}{B}\right) \right]&= \frac{1}{2} \left[ \frac{1}{2} \min (1,\exp (u_R)) + \frac{1}{2} \min (1,\exp (- u_R))\right] = {\left\{ \begin{array}{ll} \frac{1}{2} &{} \text {if } u_R = 0 \\ < \frac{1}{2} &{} \text {if } u_R \ne 0 \end{array}\right. } \end{aligned} \end{aligned}$$

Equation (7) shows that only when someone is indifferent about both alternatives $(u_R = 0)$, i.e., they are neither appealing nor unappealing, the probability of exchanging is a half. In all other cases the endowment effect rears its head and the probability of exchanging will be less a half. Having demonstrated how several choice phenomena are explained in this setup, we turn to another property of our model, response times.

### Response times

Response time predictions can be very informative when comparing different choice structures, evaluation processes and trigger conditions. As shown in the methods section, the single spin-flip algorithm provides the expected number of iterations until a choice condition is reached as a proxy for time. This can be used to investigate expected ordering of response times for a particular choice structure. For example, in a simple structure with no relationship existing between alternatives the expected number of iterations before a choice is triggered increases in the number and appeal of the alternatives. Or, assuming that longer response times are indicative of more deliberate decision-making, i.e., requiring more visits to a choice condition before a choice is triggered, we expect that context effects diminish and choices get increasingly rational. With increasing the required number of visits to a choice condition, choice probabilities go to Eq. (6) if a choice is sampled proportional to the number of visits of each condition. If the first alternative for which the choice condition has been visited the required number times is chosen, choice probabilities go to one for the alternative with the highest general appeal.

The model also allows incorporating response time phenomena such as the speed-accuracy trade-off^95^, which predicts that under time-pressure choices are faster but less accurate, through $\beta$. In an application of the Ising model to attitudes^96,97^, the attention to an attitude object is represented by $\beta$. This interpretation fits well within the choice model, as such an inverse relation can also be assumed between time-pressure and attention. As $\beta$ scales the magnitude of the entire choice structure, lower values will not only reduce the expected number of iterations before a choice is made, but also the effect of $\mathbf {A}$ and $\mathbf {b}$, and with that the magnitude of the context effects. This is also in line with research that showed that context effects tend to be smaller under time-pressure^66,98^. Choice expectations under time-pressure can be even more fine-tuned by using $\mu$. For example, the assumption that people under time-pressure only focus on the general appeal of the alternative can be modelled by letting $\mu = {}^{1}\!/_{\beta }$. In the methods section we show how different forms of time-pressure, modelled as variations in the relation between $\beta$ and $\mu$, influence the expected choice probabilities for the attraction effect.

## Discussion

In this article we proposed a model for choices in which the choice structure is represented by a network, for which the node states have a distribution known as the quadratic exponential binary distribution or Ising model. Single spin-flip dynamics describe the alternative evaluation process in our basic setup, and potential choice conditions are states in which only one alternative is active. The invariant distribution of this choice process is the same as that of several classic choice models with the property of simple scalability, which guarantees choices to be rational. Stopping when the choice conditions hold for the first time predicts a series of well known violations of rationality known as context effects and several other choice phenomena. This approach allows one to represent choice situations in an accessible way, and can be used to compare different choice structures, alternative evaluation process assumptions, and trigger variations with respect to the choice behaviour they predict. Furthermore, as we show next, it implements or can be extended with features and mechanics used in more complex choice models. We first review the relation between our model and the elimination by aspects (EBA) model, multi-alternative decision field theory (MDFT), the leaky competing accumulator model (LCA), and (simple) 2N-ary Choice Tree (2NCTs), and end with discussing some limitations and prospects of our approach.

One of the first models to offer an explanation for the similarity effect was Tversky’s EBA model^42^. In the EBA model an alternative is characterised by a collection of attributes. At each step in the choice process one attribute is selected proportional to an attention weight, and alternatives without this attribute are eliminated until only one alternative remains. Although the utility of an alternative in the EBA model is a function of unique attributes and those shared between pairs of alternatives, choice probabilities can be calculated independently of the specific attributes. The EBA model can only explain the similarity effect, which occurs when a subset of the alternatives share some attributes that are not shared with the other alternatives. For example, the two Beethoven recordings share attributes that are not shared by the Debussy recording. As such the probability of selecting an attribute that is unique to a Beethoven is smaller when both recordings are in the choice set with the Debussy, compared to when only one is.

MDFT, the LCA, and 2NCTs, are capable of explaining more context effects. They are sequential sampling models, which entails that (noisy) information about the alternatives is integrated in an accumulator for each alternative throughout the choice process. Whereas MDFT and the LCA each assume one accumulator per alternative, in the 2NCTs each alternative has two accumulators, one for positive information and one for negative information. For all models the process stops when either a time limit is reached or one of the (positive) accumulators crosses a threshold, triggering in both cases a choice for the alternative for which the most (positive) information is accumulated. In the 2NCTs the process can also terminate when for all but one of the alternatives the threshold of the negative accumulator is crossed and these alternatives are eliminated.

As in the EBA model, an alternative’s appeal is a function of its attributes and attention switches between these attributes over time. The explanation of the context effects in MDFT, the LCA, and 2NCTs rest primarily on some form of asymmetry between, or particular positioning of, the alternatives on attributes and must therefore be specified for all alternative-attribute combinations. In our model we do not need to specify different attributes or assume switching between attributes to predict context effects, as the influence of attributes is captured in the general appeal of the alternative. The influence of different attributes can be made explicit by adding an additional layer of *attribute nodes*, in which the position of the alternative on the attribute is encoded in the edge between them. During the process one can assume that attributes are always active and function as cues, or let their activity vary over time to incorporate the assumption that attention stochastically switches between attributes.

The models require several other mechanisms to explain context effects. Lateral inhibition^99^, a neural concept in which an exited neuron inhibits its neighbours, is applied with the same magnitude for all alternative pairs in the LCA, and decreasing with the distance between alternatives in MDFT. The LCA and 2NCTs (also) rely on an implementation of loss aversion. In the LCA accumulators can only take non-negative values and the influence of negative differences between attribute values relative to positive differences is reduced. In the 2NCTs the evaluation process decreases the probability of updating a negative accumulator relative to that of a positive accumulator.

Both inhibition and loss aversion are part of our model. Loss aversion is implemented within the process of single spin-flip dynamics, i.e., the probability of activating an appealing alternative is always one, whereas the probability of eliminating an appealing alternative is decreasing in the appeal. Inhibition comes in the form of the negative interactions between the alternatives and plays a vital role in the prediction of context effects. Global inhibition as found in the LCA can be implemented by lowering the interaction between each pair of alternatives with a constant. Otherwise independent alternatives then become negatively related and the evaluation process will move faster to a potential choice condition^100^.

Although our approach has several advantages with respect to the more complex multi-attribute multi-alternative models, it does not provide the same insight in choices times distributions. That being said, our predictions with respect to the ordering of response times are often the same as these models, and the model even provides novel explanations for some response time phenomena. For example, the prediction that context effects strengthen with longer decision times can, in addition to time-pressure, also be explained by the format of the experiment. The study manipulated time-pressure by letting a participants evaluate the characteristics of novel stimuli for 2, 4, 6, or 8 s, after which a choice had to be made immediately. Participants who could look to the stimuli for less than 8 s made choices less consistent with the context effects compared to participants that could look for 8 s^98^.

Whenever a choice is presented the available alternatives must be encoded to determine their appeal, a process that takes time. For daily choices alternatives are recurring and embedded within a stable choice structure such that decoding takes almost no time. New alternatives must be placed within the global structure and connected to the relevant concepts before their perceived appeal stabilises. As multi-attribute multi-alternative models must specify all alternatives-attribute combinations, studies often use fictional products defined on small numbers of attributes only. Fictional alternatives are new to the participant and time is required to derive the general appeal of, and establish the appropriate relationships between, alternatives. This explanation is consistent with a transition from $\beta = 0$, a choice structure with no relationships and no general appeal, to $\beta = 1$, a fully formed structure, during this transition the magnitude of the context effects increases. In contrast to the other models, however, our model predicts that context effects diminish when time-pressure is reduced even further and more deliberation can take place before a choice is triggered.

We discussed our model assuming that all alternatives are known and everyone has the same choice structure. It is clear that choice situations exist in which the alternatives are not necessarily provided, or in such large numbers that evaluating them all might be infeasible. Furthermore, while it is a common assumption that the behaviour of individuals can be described by a set of parameters for the group, it is often rather unrealistic. Future research should focus on extending the model for these situations. For example by introducing initial selection probabilities for alternatives to be included in the choice structure, or interpreting $\beta$ and $\mu$ as parameters that are different for each person, or extend the model for individual choice structures^101^.

We also recognise that data and findings in psychology from a few decades ago are sometimes questionable with respect to the current standards of research. For example, papers that used alternative methods to analyse data from a study by Tversky on within-person transitivity^102^, find that for several (but not all) persons for whom Tversky asserted that they showed intransitive behaviour, the results are no longer significant^103–105^. Although we believe that there is general consensus, based on a large body of research, that humans do not always make rational decisions, some experiments about irrational choice behaviour remain disputed. We therefore want to stress the importance of replicating these studies, or setting up new experiments that investigate the same phenomenon.

Another point, discussed in the paper by Regenwetter et al.^104^, is that often behaviour that may seem intransitive is actually rational when previously unobserved variables are taken into account. As an example they take a student that assumes that their supervisor’s perceived utility of meeting locations is stable over time, but that this is not the case as the varying teaching location of the supervisor actually determines this utility. As such, while the student judges the choices for meeting locations of the supervisor to be intransitive, this is in reality not the case. This example shows that, particularly for within-person choice effects, it is important to take context variables into account. In our model this could be accounted for by introducing nodes for these context variables, in case of the example a node for each of the teaching locations, that has a positive relation with the closest meeting locations, and which is active if the day of the meeting the supervisor has to teach in that location.

Even though very precise quantitative predictions are generally out of reach with behavioural data, there is merit in the prediction of qualitative phenomena, such as the ordering of probabilities, interaction effects, shapes of distributions, and even phenomena such as phase transitions. Formalising our theories about behaviour allows us to obtain these predictions. While we already propose multiple extensions to our model, ideally, it will be formal theories that dictate the assumptions, mechanisms, and structural characteristics to be used in a particular setup. By taking the opportunities to extend, refine, and improve the elements of the choice model, we can hopefully create a broad understanding of how the many phenomena and key theoretical principles from different types of decision-making are connected.

## Methods

We derive the expression for the expected choice probabilities for the single spin-flip algorithm, demonstrate these steps for the attraction effect, and provide the parameter values for all examples used in the main text. We then visualise how variations of $\beta$ and $\mu$ influence the choice probabilities for the attraction effect. We end by showing the parameter based expression for the binary case and discuss some properties with respect to rational choice.

### Single spin-flip dynamics

For a choice structure with *k* cues and *m* alternatives there are $2^m$ possible configurations of $\mathbf {x}$. We use $\mathbf {x}_i$ to denote the *i*th of these $2^m$ possible states. Let $\mathbf {P}$ be a square matrix of order $2^m$ in which element $P_{ij}$ contains the probability of transitioning from $\mathbf {x}_i$ to $\mathbf {x}_j$ in one step of the single spin-flip algorithm, and $P_{ii}$ contains the probability staying in the current state. As the algorithm changes at most one alternative at each iteration $\mathbf {P}$ will be highly sparse with at most *m* non-zero elements in each row. From $\mathbf {P}$ we obtain the expected rational choice probabilities, contained in the stationary distribution, as they are proportional to the elements of the first eigenvector of $\mathbf {P}$ that correspond to states in which the choice conditions are met (i.e., $\sum _{i=1}^n x_i = k + 1)$. We will not go into this approach at length as these probabilities can simply be obtained from the conditional distribution presented in Eq. (6).

The expected choice probabilities for stopping as soon as the choice conditions are met for the first time are obtained by reformulating the Markov chain with transition matrix $\mathbf {P}$ as an absorbing chain. To that end we make a distinction between the *m* absorbing states, i.e., those states in which only one alternative is active, and $2^m - m$ transient states, i.e., those states in which more than one alternative or no alternatives are active. The transition matrix for the absorbing chain $\mathbf {P}^{*}$ has the canonical form:

8 $$\begin{aligned} \begin{aligned} \mathbf {P}^{*}&= \begin{bmatrix} \mathbf {Q} &{} \mathbf {R} \\ \mathbf {0} &{} \mathbf {1} \end{bmatrix} \, , \end{aligned} \end{aligned}$$

in which $\mathbf {Q}$ contains the transition probabilities between transient states, $\mathbf {R}$ contains the transition probabilities from transient states to absorbing states, $\mathbf {1}$ contains the transition probabilities between absorbing states, i.e., an identity matrix of order *n*, and $\mathbf {0}$ contains the transition probabilities from absorbing states to transient states, i.e., a matrix with zeros. Rearranging $\mathbf {P}$ in its canonical form allows us to derive the expected progression of the Markov chain more easily^106^.

Let $y \in \{1,2,\dots , m\}$ denote the chosen alternative, let $\mathbf {z} = [z_1, z_2, \dots , z_{{\scriptscriptstyle {2}}^m}]$ denote the resting state probabilities in which $z_i \in [0,1]$ denotes the probability for the choice process to start in alternative configuration $\mathbf {x}_i$. We divide $\mathbf {z}$ into the probabilities for starting in an absorbing state$(\mathbf {z}_a)$, and for starting in a transient state $(\mathbf {z}_t)$. Lastly, let *t* denote the number of iterations of the Metropolis algorithm.

The (marginal) probability that alternative *y* is chosen from the set of alternatives *S* is:

9 $$\begin{aligned} \begin{aligned} p_{\scriptscriptstyle {S}}(y) = \mathbf {z}_a \, \mathbf {1}_y + \mathbf {z}_t \, \sum \limits _{t = 0}^{\infty } \, \mathbf {Q}^t \, \mathbf {R}_y \,, \end{aligned} \end{aligned}$$

in which $\mathbf {1}_y$ and $\mathbf {R}_y$ represent the *y*th column of the matrices $\mathbf {1}$ and $\mathbf {R}$ respectively. Using the property of geometric series to rewrite the infinite sum over $\mathbf {Q}^t$ this expression simplifies to:

10 $$\begin{aligned} \begin{aligned} p_{\scriptscriptstyle {S}}(y)&= \mathbf {z}_a \, \mathbf {1}_y + \mathbf {z}_t \, (\mathbf {I} - \mathbf {Q})^{-1} \, \mathbf {R}_y \,. \end{aligned} \end{aligned}$$

The expected number of Metropolis iterations before alternative *y* is chosen is:

11 $$\begin{aligned} \begin{aligned} E(\text {t} \mid y)&= \frac{1}{p_{\scriptscriptstyle {S}}(y)} \, \left[ \mathbf {z}_t \, \sum \limits _{t = 1}^{\infty } \, t \, \mathbf {Q}^{t-1} \, \mathbf {R}_y \right] \, . \end{aligned} \end{aligned}$$

Once again we can rewrite the infinite sum over $t \, \mathbf {Q}^{t-1}$ and simplify the expression to:

12 $$\begin{aligned} \begin{aligned} E(\text {t} \mid y)&= \frac{1}{p_{\scriptscriptstyle {S}}(y)} \, \left[ \mathbf {z}_t \, (\mathbf {I} - \mathbf {Q})^{-2} \, \mathbf {R}_y \right] \, . \end{aligned} \end{aligned}$$

$$\varvec{\beta }$$ and $$\varvec{\mu }$$

**Figure 7:**
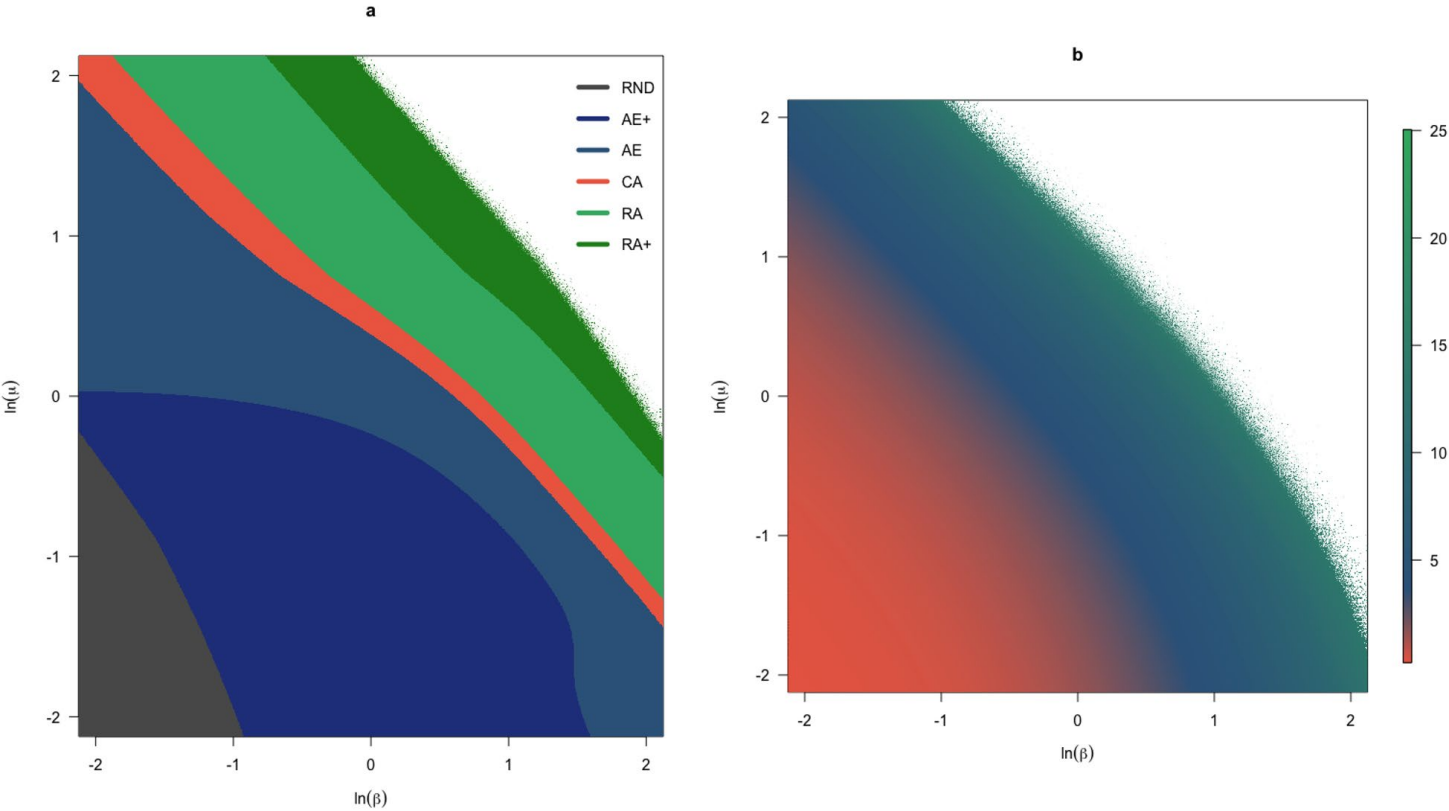
Influence of $\beta$ and $\mu$ on the choice probabilities (**a**) and response times (**b**) for the attraction effect example. The axes for both plots show $\beta$ and $\mu$ on a log-scale. (**a**) 6 different response phases are identified as a function of changes in $\beta$ and $\mu$. (**b**) Response times are approximated with the log of the expected number of iterations before termination, averaged over all three choices.

Figure 7 visualises the effect of $\beta$ and $\mu$ on the expected choice probabilities and response times for the attraction effect example from Fig. 4a. In Fig. 7a we have identified 6 different response phases for this example.

1. *Random phase (RND)* The choice probabilities for choosing the money $({\$})$, the nice pen $(P_+)$, or the not so nice pen pen $(P_+)$, differ at most $10\%$ from one another.
2. *Strong attraction effect phase (AE+)* The probability of choosing $P_+$ is greater than the probability of choosing ${\$}$
3. *Normal attraction effect phase (AE)* The probability of choosing ${\$}$ is smaller than $62\%$
4. *Choice axiom phase (CA)* The choice probabilities for all options differs only a marginally from those expected under Eq. (6) if $\beta$ and $\mu$ would both be one.
5. *Increasingly rational phase (RA)* The probability of choosing ${\$}$ is larger than $66\%$
6. *Rational phase (RA+)* The probability of choosing ${\$}$ is larger than $90\%$

The distribution of the different phases as a function of $\log (\beta )$ and $\log (\mu )$ in Fig. 7a shows that the random phase primarily takes place when both $\beta$ and $\mu$ are small. When $\mu$ is small and $\beta$ goes up the attraction effect is at its strongest, which makes sense as the influence of relationships between alternatives becomes stronger, while keeping the influence of the general appeal small. When both $\beta$ and $\mu$ increase we find that choices become increasingly rational, i.e., eventually the money is chosen almost with near certainty. At this point we run into the limit of our computational precision, as is shown by the white area in the upper right corner of both plots. Specifically, as the initial condition will go to one for all alternatives active, and the probability of transitioning out of this state becomes increasingly close to zero, calculating the inverse of the transitions matrix can no longer be done accurately.

This is also what we see when looking to the distribution of the log mean response times as a function of $\log (\beta )$ and $\log (\mu )$ in Fig. 7b, which shows clearly that these are increasing in both $\beta$ and $\mu$. While in most cases the expected number of iterations is thus small, the median of the plot range is approximately 38 iterations before a response is selected, at some point the mean expected number of iterations before a choice is made goes up to 54419290677, or fifty-four billion four hundred nineteen million two hundred ninety thousand six hundred seventy-seven. This tells us that for those corresponding values of $\beta$ and $\mu$, the probability of getting out of a transitive state is extremely small. Of course, the number of iterations is only a proxy for response times and therefore does not tell us how long the choice process will actually take. For example, if the log number of iterations would be the number of seconds a choice process takes, 54419290677 iterations would amount to less than 25 s.

Looking to both Fig. 7a,b we find that our model predicts random behaviour for very short response times, and with increasing these the context effects become visible. When response times go up even further, eventually the context effects diminish again and choices become increasingly rational. In the limit, choice probabilities go to one for the alternative with the largest general appeal and the expected number of iterations goes to infinite.

### Rational choices

Expressing the expected choice probabilities as a function of the parameters $\mathbf {A}$ and $\mathbf {b}$ for a choice with *n* alternatives, requires one to write out all the possible paths to the *n* choice conditions from all $2^n - n - 1$ configurations in which at least two alternatives are active. As this expression becomes already incomprehensible for $n=3$, we will limit ourselves to the simplest case of a binary choice problem. If we define $u_i = \sum _{k \in {\scriptscriptstyle {K}}} a_{ik} + b_i$, the probability of choosing alternative *x* over *y* in a binary choice problem is given by:

13 $$\begin{aligned} \begin{aligned} p_{x,y}(x)&= p_x + p_{\scriptscriptstyle {0}} \, \frac{1}{1 + \exp {\left[ \min (0,u_y) - \min (0,u_x) \right] }} + p_{\scriptscriptstyle {1}} \, \frac{1}{1 + \exp {\left[ \min (0,-[a_{xy} + u_x]) - \min (0,-[a_{xy} + u_y]) \right] }} \, , \end{aligned} \end{aligned}$$

in which $p_x$ denotes the probability to start in the configuration in which $x=1$ and $y=0$, as such meeting the choice conditions and directly triggering a choice for alternative *x*. $p_{\scriptscriptstyle {0}}$ and $p_{\scriptscriptstyle {1}}$ denote the probabilities to start in a configuration in which all alternatives are inactive $(p_{\scriptscriptstyle {0}})$ or active $(p_{\scriptscriptstyle {1}})$. Although this seems like a straightforward expression, even when assuming that there is no relation between the alternatives $(a_{xy} = 0)$, we already obtain the four possible formulations depending on the values for $u_x$ and $u_y$:

14 $$\begin{aligned} \begin{aligned} p_{x,y}(x)&= {\left\{ \begin{array}{ll} p_x + p_{\scriptscriptstyle {0}} \, \frac{1}{2} + p_{\scriptscriptstyle {1}} \, \frac{1}{1 + \exp {\left[ u_y - u_x\right] }} &{} \text {if } \{u_x,u_y\}> 0 \\ p_x + p_{\scriptscriptstyle {0}} \, \frac{1}{1 + \exp {\left[ u_y - u_x\right] }} + p_{\scriptscriptstyle {1}} \, \frac{1}{2} &{} \text {if } \{u_x,u_y\}< 0 \\ p_x + p_{\scriptscriptstyle {0}} \, \frac{1}{1 + \exp {\left[ u_y\right] }} + p_{\scriptscriptstyle {1}} \, \frac{1}{1 + \exp {\left[ - u_x\right] }} &{} \text {if } u_x> 0 > u_y \\ p_x + p_{\scriptscriptstyle {0}} \, \frac{1}{1 + \exp {\left[ - u_x\right] }} + p_{\scriptscriptstyle {1}} \, \frac{1}{1 + \exp {\left[ u_y\right] }} &{} \text {if } u_x< 0 < u_y \end{array}\right. } \end{aligned} \end{aligned}$$

From Eqs. (13) and (14) it becomes clear that if no relationships exist between alternatives, the probability of choosing alternative *x* is purely a function of the difference between $u_x$ and $u_y$ if we started in either $p_{\scriptscriptstyle {0}}$ or $p_{\scriptscriptstyle {1}}$. While it might seem a plausible assumption that choices will be at least weakly rational, i.e., $p_{x,y}(x) \ge {}^{1}\!/_{2} \iff u_x \ge u_y$, if there is no interaction between the alternatives, this is not necessarily the case.

For example, if the relation between cue *k* and alternative *x* is positive ($a_{kx} = 10$), but the general appeal of *x* is negative ($b_x = -5$), and the relation between cue *k* and alternative *y* is negative ($a_{ky} = 10$), but the general appeal of *y* is positive ($b_y = 5$). We find that $u_x = 5$ and $u_y = -5$, and as $u_x > u_y$ we would expect $p_{x,y}(x) \ge {}^{1}\!/_{2}$. Using Eq. (14) we can calculate that when starting in a non-absorbing configuration both transition probabilities are almost one, such that $p_{x,y}(x) \approx p_x + p_{\scriptscriptstyle {0}} + p_{\scriptscriptstyle {1}}$ and the probability of choosing *x* over *y* is the sum of all starting configurations except $p_y$, i.e., $p_{x,y}(x) \approx 1 - p_y$. However, whereas the transition probabilities are only a function of the difference between $u_x$ and $u_y$, the starting probabilities are not. In the resting state distribution the cue would almost always be inactive if $b_k<< 0$, and the probabilities $p_{\scriptscriptstyle {0}}, p_x, p_y$ and $p_{\scriptscriptstyle {1}}$ become primarily a function of $b_x$ and $b_y$. As $b_y>> b_x$, we find that $p_y \approx .99$, which implies that 99% of the time we will start in the configuration that will immediately trigger the choice for alternative *y*. Although this situation might not necessarily be encountered in real life, it shows that determining when choices are guaranteed to be even weakly rational is not straightforward.

## Supplementary information

Supplementary Information.

## References

[1] Simon HA (1955). A behavioral model of rational choice. Q. J. Econom..

[2] Simon HA (1959). Theories of decision-making in economics and behavioral science. Am. Econ. Rev..

[3] Thaler R (1980). Toward a positive theory of consumer choice. J. Econ. Behav. Org..

[4] McFadden D (2001). Economic choices. Am. Econ. Rev..

[5] Edwards W (1954). The theory of decision making. Psychol. Bull..

[6] Luce RD (1959). Individual Choice Behavior: A Theoretical Analysis.

[7] Restle F (1961). Psychology of Judgment and Choice: A Theoretical Essay.

[8] Kahneman D, Tversky A (1982). The psychology of preferences. Sci. Am..

[9] Rasch G (1960). Probabilistic Models for Some Intelligence and Attainment Tests.

[10] Lord FM, Novick MR (1968). Statistical Theories of Mental Test Scores.

[11] Busemeyer JR (1985). Decision making under uncertainty: A comparison of simple scalability, fixed-sample, and sequential-sampling models. J. Exp. Psychol. Learn. Mem. Cogn..

[12] Bogacz R, Brown E, Moehlis J, Holmes P, Cohen JD (2006). The physics of optimal decision making: A formal analysis of models of performance in two-alternative forced-choice tasks. Psychol. Rev..

[13] Ratcliff R, McKoon G (2008). The diffusion decision model: Theory and data for two-choice decision tasks. Neural Comput..

[14] Roe RM, Busemeyer JR, Townsend JT (2001). Multialternative decision field theory: A dynamic connectionst model of decision making. Psychol. Rev..

[15] Gold JI, Shadlen MN (2007). The neural basis of decision making. Annu. Rev. Neurosci..

[16] Shadlen MN, Kiani R (2013). Decision making as a window on cognition. Neuron.

[17] Awad E (2018). The moral machine experiment. Nature.

[18] Frank D-A, Chrysochou P, Mitkidis P, Ariely D (2019). Human decision-making biases in the moral dilemmas of autonomous vehicles. Sci. Rep..

[19] Pareto V (1971). Manual of Political Economy.

[20] Mill JS (1836). On the Definition of Political Economy, and of the Method of Investigation Proper to it.

[21] McFadden D, Manski CF (1981). Econometric models of probabilistic choice. Structural Analysis of Discrete Data with Econometric Applications.

[22] Von Neumann J, Morgenstern O (1944). Theory of Games and Economic Behavior.

[23] Arrow KJ (1958). Utilities, attitudes, choices: A review note. Econometrica.

[24] Luce RD, Suppes P, Luce RD, Bush RR, Galanter E (1965). Preference, utility, and subjective probability. Handbook of Mathematical Psychology.

[25] Thurstone LL (1927). A law of comparative judgment. Psychol. Rev..

[26] Block HD, Marschak J (1960). Random orderings and stochastic theories of response. Economic Information, Decision, and Prediction: Selected Essays: Volume I Part I Economics of Decision.

[27] Marschak J, Arrow K (1960). Binary choice constraints and random utility indicators. Stanford Symposium on Mathematical Methods in the Social Sciences.

[28] Becker GM, DeGroot MH, Marschak J (1963). Stochastic models of choice behavior. Behav. Sci..

[29] McFadden D, Zarembka P (1974). Conditional logit analysis of qualitative choice behavior. Frontiers in Econometrics.

[30] Bock RD (1972). Estimating item parameters and latent ability when responses are scored in two or more nominal categories. Psychometrika.

[31] Boltzmann L (1877). Über die beziehung zwischen dem zweiten hauptsatze des mechanischen wärmetheorie und der wahrscheinlichkeitsrechnung, respective den sätzen über das wärmegleichgewicht.

[32] Gibbs J (1902). Elementary Principles of Statistical Mechanics.

[33] Bradley RA, Terry ME (1952). Rank analysis of incomplete block designs: I. The method of paired comparisons. Biometrika.

[34] Zermelo E (1929). Die berechnung der turnier-ergebnisse als ein maximumproblem der wahrscheinlichkeitsrechnung. Math. Z..

[35] Coombs CH (1958). On the use of inconsistency of preferences in psychological measurement. J. Exp. Psychol..

[36] Tversky A, Russo J (1969). Similarity and substitutability in binary choice. J. Math. Psychol..

[37] Evans NJ, Holmes WR, Trueblood JS (2019). Response-time data provide critical constraints on dynamic models of multi-alternative, multi-attribute choice. Psychon. Bull. Rev..

[38] Debreu G (1960). Review of Individual choice behavior: A theoretical analysis by R. Duncan Luce. American Economic Review.

[39] Becker GM, Degroot MH, Marschak J (1963). Probabilities of choices among very similar objects: An experiment to decide between two models. Behav. Sci..

[40] Krantz DH (1967). Rational distance functions for multidimensional scaling. J. Math. Psychol..

[41] Rumelhart DL, Greeno JG (1971). Similarity between stimuli: An experimental test of the Luce and Restle choice models. J. Math. Psychol..

[42] Tversky A (1972). Elimination by aspects: A theory of choice. Psychol. Rev..

[43] Luce RD (1977). The choice axiom after twenty years. J. Math. Psychol..

[44] Huber J, Payne JW, Puto C (1982). Adding asymmetrically dominated alternatives: Violations of regularity and the similarity hypothesis. J. Consumer Res..

[45] Simonson I (1989). Choice based on reasons: The case of attraction and compromise effects. J. Consumer Res..

[46] Simonson I, Tversky A (1992). Choice in context: Tradeoff contrast and extremeness aversion. J. Mark. Res..

[47] Tversky A, Simonson I (1993). Context-dependent preferences. Manage. Sci..

[48] Trueblood JS, Brown SD, Heathcote A, Busemeyer JR (2013). Not just for consumers: Context effects are fundamental to decision making. Psychol. Sci..

[49] Trueblood JS (2012). Multialternative context effects obtained using an inference task. Psychon. Bull. Rev..

[50] Pettibone JC, Wedell DH (2007). Testing alternative explanations of phantom decoy effects. J. Behav. Decis. Making.

[51] Pettibone JC, Wedell DH (2000). Examining models of nondominated decoy effects across judgment and choice. Org. Behav. Hum. Decis. Process..

[52] Pratkanis AR, Farquhar PH (1992). A brief history of research on phantom alternatives: Evidence for seven empirical generalizations about phantoms. Basic Appl. Soc. Psychol..

[53] Simon HA (1980). The behavioral and social sciences. Science.

[54] Kahneman D, Tversky A (1983). Choices, values, and frames. Am. Psychol..

[55] Tversky A, Kahneman D (1986). Rational choice and the framing of decisions. J. Bus..

[56] Busemeyer JR, Townsend JT (1992). Fundamental derivations from decision field theory. Math. Soc. Sci..

[57] Busemeyer JR, Townsend JT (1993). Decision field theory: A dynamic-cognitive approach to decision making in an uncertain environment. Psychol. Rev..

[58] Busemeyer JR, Diederich A (2002). Survey of decision field theory. Math. Soc. Sci..

[59] Hotaling J, Busemeyer J, Li J (2010). Theoretical developments in decision field theory: Comment on Tsetsos, Usher, and Chater (2010). Psychol. Rev..

[60] Berkowitsch NA, Scheibehenne B, Rieskamp J (2014). Rigorously testing multialternative decision field theory against random utility models. J. Exp. Psychol. Gen..

[61] Usher M, McClelland JL (2001). The time course of perceptual choice: The leaky, competing accumulator model. Psychol. Rev..

[62] Usher M, McClelland JL (2004). Loss aversion and inhibition in dynamical models of multialternative choice. Psychol. Rev..

[63] Bogacz R, Usher M, Zhang J, McClelland JL (2007). Extending a biologically inspired model of choice: Multi-alternatives, nonlinearity and value-based multidimensional choice. Philos. Trans. R. Soc. B Biol. Sci..

[64] Tsetsos K, Usher M, Chater N (2010). Preference reversal in multiattribute choice. Psychol. Rev..

[65] Brown SD, Heathcote A (2008). The simplest complete model of choice response time: Linear ballistic accumulation. Cogn. Psychol..

[66] Trueblood JS, Brown SD, Heathcote A (2014). The multiattribute linear ballistic accumulator model of context effects in multialternative choice. Psychol. Rev..

[67] Holmes WR, Trueblood JS, Heathcote A (2016). A new framework for modeling decisions about changing information: The piecewise linear ballistic accumulator model. Cogn. Psychol..

[68] Wollschläger LM, Diederich A (2012). The 2n-ary choice tree model for n-alternative preferential choice. Front. Psychol..

[69] Wollschläger, L. M. & Diederich, A. A computational model for constructing preferences for multiple choice options. in Gunzelmann, G., Howes, A., Tenbrink, T. & Davelaar, E. (eds.) *Proceedings of the 39th Annual Conference of the Cognitive Science Society*, 1351–1356 (Austin, 2017).

[70] Bhatia S (2013). Associations and the accumulation of preference. Psychol. Rev..

[71] Turner BM, Schley DR, Muller C, Tsetsos K (2018). Competing theories of multialternative, multiattribute preferential choice. Psychol. Rev..

[72] Wollschlaeger LM, Diederich A (2020). Similarity, attraction, and compromise effects: Original findings, recent empirical observations, and computational cognitive process models. Am. J. Psychol..

[73] Lenz W (1920). Beitrag zum verstandnis der magnetischen erscheinunge in festen korpern. Phys. Z..

[74] Ising E (1925). Beitrag zur theorie des ferromagnetismus. Z. für Phys. A Hadrons Nuclei.

[75] Niss M (2005). History of the Lenz-Ising model 1920–1950: From ferromagnetic to cooperative phenomena. Arch. Hist. Exact Sci..

[76] Cox DR (1972). The analysis of multivariate binary data. Appl. Stat..

[77] Cox DR, Wermuth N (1994). A note on the quadratic exponential binary distribution. Biometrika.

[78] Marsman M (2018). An introduction to network psychometrics: Relating Ising network models to item response theory models. Multivar. Behav. Res..

[79] Fierst JL, Phillips PC (2015). Modeling the evolution of complex genetic systems: The gene network family tree. J. Exp. Zool. B.

[80] Marsman M, Maris G, Bechger T, Glas C (2015). Bayesian inference for low-rank Ising networks. Sci. Rep..

[81] Van Der Maas HLJ (2006). A dynamical model of general intelligence: The positive manifold of intelligence by mutualism. Psychol. Rev..

[82] Kruis J, Maris G (2016). Three representations of the Ising model. Sci. Rep..

[83] Epskamp S, Maris G, Waldorp LJ, Borsboom D, Irwing P, Booth T, Hughes DJ (2018). Network psychometrics. The Wiley Handbook of Psychometric Testing: A Multidisciplinary Reference on Survey, Scale and Test Development.

[84] Galam S, Moscovici S (1991). Towards a theory of collective phenomena: Consensus and attitude changes in groups. Eur. J. Soc. Psychol..

[85] Galam S (1997). Rational group decision making: A random field Ising model at T= 0. Physica A.

[86] Verdonck S, Tuerlinckx F (2014). The Ising decision maker: A binary stochastic network for choice response time. Psychol. Rev..

[87] Newman M, Barkema G (1999). Monte Carlo Methods in Statistical Physics.

[88] R Core Team. *R: A Language and Environment for Statistical Computing*. R Foundation for Statistical Computing, Vienna, Austria (2019).

[89] Aaker J (1991). The negative attraction effect? A study of the attraction effect under judgment and choice. Adv. Consumer Res..

[90] Frederick S, Lee L, Baskin E (2014). The limits of attraction. J. Mark. Res..

[91] Simonson I (2014). Vices and virtues of misguided replications: The case of asymmetric dominance. J. Mark. Res..

[92] Spektor MS, Kellen D, Hotaling JM (2018). When the good looks bad: An experimental exploration of the repulsion effect. Psychol. Sci..

[93] Kahneman D, Knetsch JL, Thaler RH (1990). Experimental tests of the endowment effect and the coase theorem. J. Polit. Econ..

[94] Mather M, Johnson MK (2000). Choice-supportive source monitoring: Do our decisions seem better to us as we age?. Psychol. Aging.

[95] Luce RD (1986). Response Times: Their Role in Inferring Elementary Mental Organization.

[96] Dalege J, Borsboom D, van Harreveld F, van der Maas HL (2018). The attitudinal entropy (AE) framework as a general theory of individual attitudes. Psychol. Inquiry.

[97] van der Maas HL, Dalege J, Waldorp LJ (2020). The polarization within and across individuals: The hierarchical Ising opinion model. J. Complex Netw..

[98] Pettibone JC (2012). Testing the effect of time pressure on asymmetric dominance and compromise decoys in choice. Judgm. Decis Making.

[99] Feldman JA, Ballard DH (1982). Connectionist models and their properties. Cogn. Sci..

[100] Yuille AL, Grzywacz NM (1989). A winner-take-all mechanism based on presynaptic inhibition feedback. Neural Comput..

[101] Marsman, M. The idiographic Ising model (2019). http://www.psyarxiv.com/h3ka5.

[102] Tversky A (1969). Intransitivity of preferences. Psychol. Rev..

[103] Iverson G, Falmagne J-C (1985). Statistical issues in measurement. Math. Soc. Sci..

[104] Regenwetter M, Dana J, Davis-Stober CP (2011). Transitivity of preferences. Psychol. Rev..

[105] McCausland WJ, Davis-Stober C, Marley A, Park S, Brown N (2020). Testing the random utility hypothesis directly. Econ. J..

[106] Diederich A, Busemeyer JR (2003). Simple matrix methods for analyzing diffusion models of choice probability, choice response time, and simple response time. J. Math. Psychol..

