# Supplementary material for: Deviations of rational choice: an integrative explanation of the endowment and several context effects
Source: Sci Rep. 2020 Oct 1;10:16226. doi: 10.1038/s41598-020-73181-2 (PMC7529946; doi:10.1038/s41598-020-73181-2)
Supplement: Supplementary file 1 — Supplementary Information. [file 41598_2020_73181_MOESM1_ESM.pdf]

Deviations of rational choice: An integrative explanation of the endowment and several context effects

Supplementary Materials

Joost Kruis[1,*], **Gunter Maris**[2], **Maarten Marsman**[1], **Maria Bolsinova**[2], **and Han L. J. van der Maas**[1]

[1]University of Amsterdam, Psychological Methods Dep., Nieuwe Achtergracht 129B, Amsterdam, 1018WS, NL
[2]ACT-Next by ACT, 500 ACT Drive, Iowa City, IA 52245, USA
[*]j.kruis@uva.nl

Single spin-flip dynamics: The attraction effect

We demonstrate how the steps from the methods section on single spin-flip dynamics are applied to obtain the results from the attraction effect as discussed in the results section and shown in Fig. 4**a** of the main text. As both β and μ were set to one for the examples, we start by defining the parameters for the connectivity matrix $\mathbf{A}$ and general appeal vector $\mathbf{b}$:

$$\mathbf{A} = \begin{array}{c} \\ R \\ \$ \\ P_+ \\ P_- \end{array} \begin{array}{c} \begin{array}{cccc} R & \$ & P_+ & P_- \end{array} \\ \begin{pmatrix} 0 & 1 & 1 & 1 \\ 1 & 0 & 0 & -2.75 \\ 1 & 0 & 0 & 0 \\ 1 & -2.75 & 0 & 0 \end{pmatrix} \end{array} \quad \mathbf{b} = \begin{array}{c} \begin{array}{cccc} b_R & b_\$ & b_{P_+} & b_{P_-} \end{array} \\ \begin{pmatrix} 0 & 4.25 & 3.675 & .825 \end{pmatrix} \end{array}. \tag{1}$$

As the choice contains three alternatives, there exist $2^3 = 8$ possible activity configurations of the alternatives $(\mathbf{x})$:

$$\mathbf{x} = \begin{array}{c} \\ x_{000} \\ x_{100} \\ x_{010} \\ x_{001} \\ x_{110} \\ x_{101} \\ x_{011} \\ x_{111} \end{array} \begin{array}{c} \begin{array}{ccc} \$ & P_+ & P_- \end{array} \\ \begin{pmatrix} 0 & 0 & 0 \\ 1 & 0 & 0 \\ 0 & 1 & 0 \\ 0 & 0 & 1 \\ 0 & 1 & 1 \\ 1 & 0 & 1 \\ 1 & 1 & 0 \\ 1 & 1 & 1 \end{pmatrix} \end{array}, \tag{2}$$

in which x_{100} represents the choice condition for choosing the money, x_{010} for choosing the nice pen, and x_{001} for choosing the plain pen.

To calculate the expected choice probabilities for each of the three rewards we need the the probability distribution over the starting configurations of both the transitive $(\mathbf{z}_t)$ and absorbing $(\mathbf{z}_a)$ states. This distribution can be obtained by calculating the expression as given in Equation 4 of the main text, for each of the 2^4 possible configurations of the nodes in the choice structure (1 cue (x_k) + 3 alternatives) and sum over those states in which the alternatives have the same configuration. To show the influence of the negative relation between \$ and P_-, we do this for both the case with (AE), and without (~~AE~~), a negative edge between them, and obtain the following probabilities:

$$
\mathbf{z}_t = \begin{array}{c} \\ x_{000} \\ x_{110} \\ x_{101} \\ x_{011} \\ x_{111} \end{array}
\begin{array}{c} \begin{array}{cc} AE & \cancel{AE} \end{array} \\ \begin{pmatrix} 0 & 0 \\ .704 & .146 \\ .003 & .008 \\ .023 & .005 \\ .258 & .838 \end{pmatrix} \end{array}
\qquad
\mathbf{z}_a = \begin{array}{c} \\ x_{100} \\ x_{010} \\ x_{001} \end{array}
\begin{array}{c} \begin{array}{cc} AE & \cancel{AE} \end{array} \\ \begin{pmatrix} .008 & .002 \\ .004 & .001 \\ 0 & 0 \end{pmatrix} \end{array}
\tag{3}
$$

From these probabilities can be seen that a negative edge between the money and the plain pen, primarily makes the plain pen much less likely to be active in the initial configuration. The 8×8 non-absorbing transition matrix $\mathbf{P}$, that represents the probabilities of switching between different configurations of the alternatives as a function of the single spin-flip algorithm, is given by:

$$
\mathbf{P} \approx
\begin{array}{c|cccccccc}
 & x_{000} & x_{100} & x_{010} & x_{001} & x_{110} & x_{101} & x_{011} & x_{111} \\
\hline
x_{000} & 0 & 0.333 & 0.333 & 0.333 & 0 & 0 & 0 & 0 \\
x_{100} & 0.002 & 0.533 & 0 & 0 & 0.333 & 0.132 & 0 & 0 \\
x_{010} & 0.003 & 0 & 0.330 & 0 & 0.333 & 0 & 0.333 & 0 \\
x_{001} & 0.054 & 0 & 0 & 0.280 & 0 & 0.333 & 0.333 & 0 \\
x_{110} & 0 & 0.003 & 0.002 & 0 & 0.863 & 0 & 0 & 0.132 \\
x_{101} & 0 & 0.333 & 0 & 0.027 & 0 & 0.306 & 0 & 0.333 \\
x_{011} & 0 & 0 & 0.054 & 0.003 & 0 & 0 & 0.610 & 0.333 \\
x_{111} & 0 & 0 & 0 & 0 & 0.333 & 0.003 & 0.027 & 0.636
\end{array}
\tag{4}
$$

We can obtain the absorbing transition matrix $\mathbf{P}^*$, that applies the condition that the process stops as soon as one of the choice conditions $(x_{100}, x_{010}, x_{001})$ is met for the first time, by setting all cells in the corresponding rows to 0 and only the diagonals to 1. As such, when the process visits one of the states, they cannot transition out of it. We can then rewrite $\mathbf{P}^*$ in its canonical form and obtain the following sub-matrices:

$$
\mathbf{Q} \approx
\begin{array}{c|ccccc}
 & x_{000} & x_{110} & x_{101} & x_{011} & x_{111} \\
\hline
x_{000} & 0 & 0 & 0 & 0 & 0 \\
x_{110} & 0 & 0.863 & 0 & 0 & 0.132 \\
x_{101} & 0 & 0 & 0.306 & 0 & 0.333 \\
x_{011} & 0 & 0 & 0 & 0.610 & 0.333 \\
x_{111} & 0 & 0.333 & 0.003 & 0.027 & 0.636
\end{array}
\qquad
\mathbf{R} \approx
\begin{array}{c|ccc}
 & x_{100} & x_{010} & x_{001} \\
\hline
x_{000} & 0.333 & 0.333 & 0.333 \\
x_{110} & 0.003 & 0.002 & 0 \\
x_{101} & 0.333 & 0 & 0.027 \\
x_{011} & 0 & 0.054 & 0.003 \\
x_{111} & 0 & 0 & 0
\end{array}
$$

$$
\mathbf{0} =
\begin{array}{c|ccccc}
 & x_{000} & x_{110} & x_{101} & x_{011} & x_{111} \\
\hline
x_{100} & 0 & 0 & 0 & 0 & 0 \\
x_{010} & 0 & 0 & 0 & 0 & 0 \\
x_{001} & 0 & 0 & 0 & 0 & 0
\end{array}
\qquad
\mathbf{1} =
\begin{array}{c|ccc}
 & x_{100} & x_{010} & x_{001} \\
\hline
x_{100} & 1 & 0 & 0 \\
x_{010} & 0 & 1 & 0 \\
x_{001} & 0 & 0 & 1
\end{array}
\tag{5}
$$

Although the steps for obtaining the inverse of the matrix $(\mathbf{I} - \mathbf{Q})^{-1}$ can be written out for the three alternative case, we will not do so here and instead just provide the outcome:

$$
(\mathbf{I} - \mathbf{Q})^{-1} \approx
\begin{pmatrix}
1 & 0 & 0 & 0 & 0 \\
0 & .137 & 0 & 0 & -.132 \\
0 & 0 & .694 & 0 & -.333 \\
0 & 0 & 0 & .390 & -.333 \\
0 & -.333 & -.003 & -.027 & .364
\end{pmatrix}^{-1}
\approx
\begin{pmatrix}
1 & 0 & 0 & 0 & 0 \\
0 & 142 & 0.2 & 3.9 & 55.4 \\
0 & 67.1 & 1.6 & 1.9 & 27.6 \\
0 & 119.3 & 0.2 & 6 & 49 \\
0 & 139.7 & 0.3 & 4 & 57.4
\end{pmatrix}
\tag{6}
$$

The probability that each reward will be chosen when the process started in a non absorbing state is given by:

$$\mathbf{z}_t\,(\mathbf{I}-\mathbf{Q})^{-1}\,\mathbf{R} \approx \begin{array}{c} x_{000} \\ x_{110} \\ x_{101} \\ x_{011} \\ x_{111} \end{array} \begin{pmatrix} 0 \\ .704 \\ .003 \\ .023 \\ .258 \end{pmatrix} \overset{\begin{array}{ccccc} x_{000} & x_{110} & x_{101} & x_{011} & x_{111} \end{array}}{\begin{pmatrix} 1 & 0 & 0 & 0 & 0 \\ 0 & 142 & 0.2 & 3.9 & 55.4 \\ 0 & 67.1 & 1.6 & 1.9 & 27.6 \\ 0 & 119.3 & 0.2 & 6 & 49 \\ 0 & 139.7 & 0.3 & 4 & 57.4 \end{pmatrix}} \overset{\begin{array}{ccc} x_{100} & x_{010} & x_{001} \end{array}}{\begin{pmatrix} 0.333 & 0.333 & 0.333 \\ 0.003 & 0.002 & 0 \\ 0.333 & 0 & 0.027 \\ 0 & 0.054 & 0.003 \\ 0 & 0 & 0 \end{pmatrix}} \tag{7}$$

$$\approx \overset{\begin{array}{ccc} x_{100} & x_{010} & x_{001} \end{array}}{\begin{pmatrix} 0.515 & 0.453 & 0.019 \end{pmatrix}}$$

We obtain the choice probabilities for each of the three rewards for the full process by adding the probability that the process already starts in an absorbing state.

$$\mathbf{z}_a\,\mathbf{1}+\mathbf{z}_t\,(\mathbf{I}-\mathbf{Q})^{-1}\,\mathbf{R} \approx \overset{\begin{array}{ccc} x_{100} & x_{010} & x_{001} \end{array}}{\begin{pmatrix} 0.008 & 0.004 & 0 \end{pmatrix}} + \overset{\begin{array}{ccc} x_{100} & x_{010} & x_{001} \end{array}}{\begin{pmatrix} 0.515 & 0.453 & 0.019 \end{pmatrix}} = \overset{\begin{array}{ccc} \$ & P_{+} & P_{-} \end{array}}{\begin{pmatrix} 0.523 & 0.457 & 0.019 \end{pmatrix}} \tag{8}$$

Choice Structures

The interaction matrix **A** and general appeal vector **b** for the similarity, compromise and phantom decoy effect are provided here.

Similarity: Figure 3 of main text

$$\mathbf{A} = \begin{array}{c} R \\ B_F \\ B_K \\ D_C \end{array} \overset{\begin{array}{cccc} R & B_F & B_K & D_C \end{array}}{\begin{pmatrix} 0 & 1 & 1 & 1 \\ 1 & 0 & -10 & 0 \\ 1 & -10 & 0 & 0 \\ 1 & 0 & 0 & 0 \end{pmatrix}} \qquad \mathbf{b} = \overset{\begin{array}{cccc} b_R & b_{B_F} & b_{B_K} & b_{D_C} \end{array}}{\begin{pmatrix} 0 & 3 & 3 & 3.405 \end{pmatrix}} \tag{9}$$

Compromise: Figure 5 of main text

$$\mathbf{A} = \begin{array}{c} C \\ L \\ M \\ H \end{array} \overset{\begin{array}{cccc} C & L & M & H \end{array}}{\begin{pmatrix} 0 & 1 & 1 & 1 \\ 1 & 0 & 0 & -15 \\ 1 & 0 & 0 & 0 \\ 1 & -15 & 0 & 0 \end{pmatrix}} \qquad \mathbf{b} = \overset{\begin{array}{cccc} b_C & b_L & b_M & b_H \end{array}}{\begin{pmatrix} 0 & 7.5 & 7.5 & 7.5 \end{pmatrix}} \tag{10}$$

Phantom: Figure 6 of main text

$$\mathbf{A} = \begin{array}{c} PC \\ D \\ R \\ T \end{array} \overset{\begin{array}{cccc} PC & D & R & T \end{array}}{\begin{pmatrix} 0 & 1 & 1 & 1 \\ 1 & 0 & [-1.5,-12] & 0 \\ 1 & [-1.5,-12] & 0 & 0 \\ 1 & 0 & 0 & 0 \end{pmatrix}} \qquad \mathbf{b} = \overset{\begin{array}{cccc} b_{PC} & b_D & b_R & b_T \end{array}}{\begin{pmatrix} 0 & 10 & 1 & 1 \end{pmatrix}} \tag{11}$$

The two values between the brackets in the connectivity matrix **A** represent the required interactions between the decoy and the rival to elicit the phantom decoy effect, given that the unavailability of the decoy is respectively unknown or known before the choice is made.
